# Physical Attributes of Tree Holes in the Atlantic Forest Edges: Evaluating Their Association with the Presence and Abundance of Immature *Haemagogus leucocelaenus*

**DOI:** 10.3390/tropicalmed8070337

**Published:** 2023-06-25

**Authors:** Rosa Maria Tubaki, Regiane Maria Tironi de Menezes, Mariana Rocha David, Raquel Gardini Sanches Palasio, Osny Tadeu de Aguiar, João Batista Baitello, Vagner Oliveira Santos, Natália Balbino, Francisco Chiaravalloti-Neto

**Affiliations:** 1 Laboratório de Entomologia Médica, Instituto Pasteur da Secretaria Estadual de Saúde de São Paulo, São Paulo 01027-000, Brazil; rmtironi@pasteur.saude.sp.gov.br; 2 Laboratório de Mosquitos Transmissores de Hematozoários, Instituto Oswaldo Cruz, Rio de Janeiro 17700-000, Brazil; maridavid@ioc.fiocruz.br; 3 Departamento de Epidemiologia, Faculdade de Saúde Pública, Universidade de São Paulo, São Paulo 05508-090, Brazil; raquelpalasio@alumni.usp.br (R.G.S.P.); franciscochiara@usp.br (F.C.-N.); 4 Instituto de Pesquisas Ambientais, Secretaria de Infraestrutura e Meio Ambiente, São Paulo 90690-000, Brazil; tadeu@sp.gov.br (O.T.d.A.); baitello@sp.gov.br (J.B.B.); 5 Superintendência de Controle de Endemias, Secretária Estadual da Saúde, São Paulo 74605-110, Brazil; vagneroliveirasantos@yahoo.com (V.O.S.); natalia.balbinoo@gmail.com (N.B.)

**Keywords:** *Haemagogus leucocelaenus*, Atlantic Forest, urban forest, forest edges, tree holes, yellow fever

## Abstract

Sylvatic yellow fever (SYF) was recently a health issue in Brazil (2016–2019) because transmission was facilitated by a high density of vectors, amplifying hosts, and low vaccine coverage of the human population, especially in urban forests in the Southeast Region of Brazil. Moreover, urban forest edges are more likely to have contact between human and sylvatic vector mosquito populations. Here, we show the association between abiotic and biotic features of tree holes as *Haemagogus leucocelaenus* rearing sites in Cantareira State Park in Atlantic Forest edges. The analyzed physical features of the tree holes were diameter at breast height, tree hole opening diameter, depth, trunk diameter, tree hole volume, collected volume, height (varying from 0.02 to 4.2 m above ground), and the presence of Culicidae species other than *Hg. leucocelaenus*. We analyzed 105 positive and 68 negative water samples for larval presence and found no differences between them, suggesting the lack of specific physical characteristics in these categories. *Hg. leucocelaenus* larval abundance was correlated with the collected volume and opening diameter of tree holes. The tree species that most represented negative breeding sites were *Euplassa cantareirae*, *Guarea macrophylla*, *Psychotria suterella*, and *Tibouchina pulchra*. Four significant clusters as areas with a high risk of SYV were identified by Get-Ordis spatial analysis. Although *Hg. leucocelaenus* larvae were found in tree holes with high water levels, their occurrence was regulated by that of other mosquito species. Our findings contribute to clarifying immature vector ecology in tree holes related to human exposure to SYF in urban forest edges.

## 1. Introduction

Yellow fever is an endemic flavivirus in Brazil, where it occurs as irregular outbreaks and transmission is driven by climate, a high density of vectors, amplifying hosts, and low vaccination coverage of the human population [1]. The most extraordinary epidemics of sylvatic yellow fever in Brazil occurred from 2016 to 2019; unlike previous occurrences, it spread in regions that were not endemic or at risk. The population living in these areas, mainly in high metropolises in Southeastern Brazil, was not immunized against yellow fever. The states of São Paulo and Rio de Janeiro were highly affected, with large numbers of human cases and deaths due to yellow fever in humans and non- human primates [2].

One of the largest urban forests in the world, Cantareira State Park is highly fragmented by urbanization. Most of the fragments’ limits change abruptly from sylvatic to urban habitat without transition, increasing the contact between human and sylvatic animal populations. The forest edge is a boundary between the habitats constituted by tree species and urban places. There are many types of container habitats [3] (Bates, 1949); however, the natural containers can be found in the forest, including rock holes, ground containers and tree holes. Tree holes can be colonized by vertebrates such as birds, bats, and arthropods [4]. Many of these are habitat specialists that require specific conditions to live. Among the Aedini tribe, a mosquito species ovipositing in tree holes and bamboo-exposed internodes [5]—*Haemagogus leucocelaenus* (Dyar and Shannon, 1924)—is the most widespread sylvatic yellow fever vector species in Brazil [6,7,8,9]. It belongs to a group of sylvatic mosquitoes whose spatial distribution is limited by habitat preference [10,11]. The seasonal dynamics of the wild vector *Hg. leucocelaenus* was previously described by using standardized ovitraps in the field to estimate vector abundance [12,13,14,15,16]. There is substantial information about the epidemiological aspects of yellow fever (e.g., vectors, routes, climate and ecological factors) with respect to the distribution of YFV primary vectors and other mosquito species that play a secondary role in transmission [17,18,19]. However, little is known about *Hg. leucocelaenus*’s bionomics and distribution in tree holes in the Atlantic Forest. Here, we aimed to investigate the associations between the abiotic and biotic characteristics of tree holes and the presence and abundance of *Hg. leucocelaenus* larvae. Our aim was to provide information that may help predict the distribution of *Hg. leucocelaenus* in urban Atlantic Forest edges. 

## 2. Materials and Methods

### 2.1. Study Area and Tree Hole Selection

**Figure 1:**
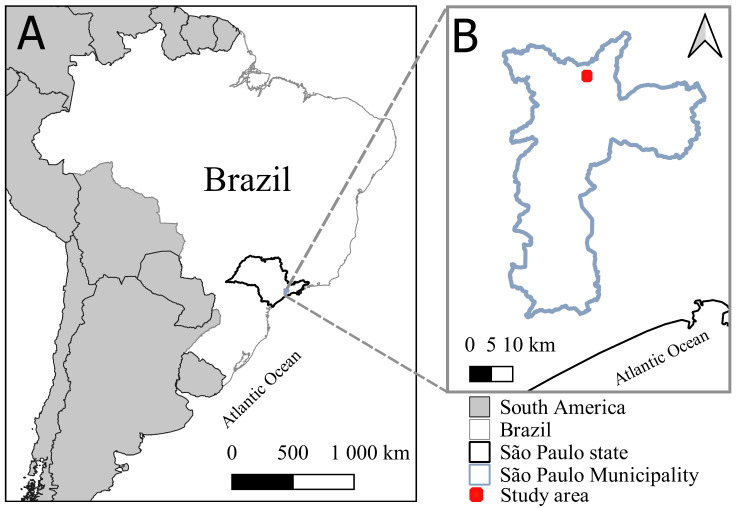
(**A**) State of São Paulo, Brazil, South America. (**B**) Study area in the municipality of São Paulo.

Cantareira State Park is situated in the northern Metropolitan Region of São Paulo city (MRSP) [20]. The MRSP is the most urbanized and populated area in the Southeast Region of Brazil [21]. Cantareira State Park is a conservation area of 80 km^2^, where forest is intermingled with urban space. It was opened to the public in 1962. Six municipalities—Caieiras, Cajamar, Francisco Morato, Franco da Rocha, Mairiporã, and São Paulo—are located on the southern border of Cantareira State Park. The MRSP is in a transitional zone between the climates Cwa (humid subtropical with dry winters and hot summers) and Af (tropical without dry season) [22,23]. Field collections were conducted at the forest edges (from 23°28′01.4″ S, 46°38′51.5″ W to 23°26′44.2″ S, 46°37′44.9″ W) that were surrounded by urban habitat. The study area was in the immediate vicinity of the urban habitats of São Paulo County (Figure 1). Tree holes were sampled at both edges of the forest. Forest edges were areas within 100 m from the interior. We moved away from trails, exploring areas of nearly 100 m^2^ from the interior to search for climbable trees and tree holes. Most tree holes were rot cavities (penetrating the wood of the tree). It is noteworthy that the vegetation of the Cantareira Forest edge is in advanced successional stage, where the average diameter at breast height (DBH) is above 20 cm and the average height of trees is more than 10 m [24,25]. 

### 2.2. Mosquito Egg and Larval Sampling

Tree holes were tested for their ability to retain water, and their internal dimensions (depth and volume), position (height) on the tree, and the DBH of the tree were measured. The selection of undamaged tree holes that could retain water was conducted in two surveys from August 2016 to July 2017. Larval collections were performed fortnightly in the same tree holes from August 2017 to July 2018. Pumps were used to completely remove water and measure the volume at the sampling time. The tree holes were refilled with water from a spring in the vicinity to maintain their original levels. The water samples were labeled in 300 mL plastic bottles and taken to the laboratory at the Instituto de Pesquisas Ambientais to identify mosquito larvae species. Mosquito larvae were identified using taxonomic keys from Arnell (1973), Forattini (2002), and Lane (1953) [10,26,27]. 

Tree holes were also explored to survey mosquito egg abundance, recorded as the cumulative number of eggs sampled fortnightly. Debris and water were removed from the tree holes, and eggs were searched and hatched in the laboratory to identify mosquito species. Samples of eggs obtained from the same tree hole were left to hatch and maintained until the adult stage. Monthly rainfall and temperature data for Cantareira State Park were obtained from the Tremembé-Jaçanã meteorological station (23°27′39″ S, 46°37′20″ W).

We used Epicollect5 [28] to collect larval and tree hole data in the field.

### 2.3. Data Analysis

We completely removed the water and debris to estimate the sample size with eggs and larvae in the tree holes. We tested the effect of the removal of water with eggs, larvae, and detritus on *Hg. leucocelaenus* larval sampling using the chi-squared test.

Statistical analysis was conducted in R version 4.1.0. [29]. The physical and biotic features of the tree holes measured for the analysis included DBH, tree hole opening diameter (DOP), depth (DEP), trunk diameter (TD), tree hole volume (THV), collected volume (CV), height above ground (HAG), rainfall, and the presence of Culicidae species other than *Hg. leucocelaenus* (OTHER_SP). DBH is the diameter of the tree trunk measured at breast height, which is a standard for measuring trees and their growth and age. Trunk diameter was estimated by measuring the tree at breast height and dividing the circumference by π=3.14, while DBH for trees with multiple stems was determined by taking the square root of the sum of all squared stem DBHs. The DOP was measured as the maximum external length of the aperture of the tree hole. The THV refers to tree hole capacity, and the CV corresponds to the water retained in tree holes that would facilitate eggs hatching. The dependent variable was mosquito species abundance. 

Tree hole samples were classified as positive or negative according to the presence or absence, respectively, of *Hg. leucocelaenus.* Because the same trees were sampled several times and most of the physical parameters were fixed over time (i.e., DBH, DOP, DEP, and THV), only samples from tree holes that did not contain *Hg. leucocelaenus* larvae were included in the “negative” group. Moreover, only samples taken when the tree hole had some water were considered. We first used principal component analysis (PCA) to explore whether positive and negative samples exhibited specific physical profiles. Principal component analysis was applied to the correlation matrix because the variables were on different scales and exhibited different variances. Therefore, the variables were standardized to mean and variance equal to zero and one, respectively. The physical parameter THV was removed from further analyses because of its strong correlation (r > 0.8) with CV.

The association between *Hg. leucocelaenus* occupancy in tree holes and habitat characteristics was investigated through a generalized linear model (GLM) with binomial distribution, also known as logistic regression, with the presence or absence of larvae as the binary dependent variable and the physical and biotic parameters DBH, DOP, DEP, CV, HAG, RAINFALL, and OTHER_SP as the independent variables. The most informative and parsimonious model was determined through stepwise model selection by second-order Akaike information criterion scores. Collinearity between independent variables was checked in the best model through variance inflation factors [30]. The assumptions of the best model were examined by checking for heteroscedasticity, the dispersion of residuals, and the presence of outliers using the R package DHARMa [31]. The McFadden’s pseudo-R2 was calculated using the “RsqGLM” function from the “modEvA” package [32] was employed as a goodness-of-fit metric. Values between 0.2 and 0.4 usually indicate a good model fit for binary data [33]. The effect of independent variables on the probability of finding *Hg. leucocelaenus* in the tree holes was considered significant when α = 0.1 and was expressed as odds ratios (ORs). Finally, the presence of *Hg. leucocelaenus* larvae was also explored across tree species. We tested whether the tree species were equally distributed between positive and negative tree hole samples by performing a chi-squared test. The contribution of each tree species’ residuals for the total chi-squared score was used as a measure of the discrepancy between the expected and observed values in the positive and negative tree hole samples. 

To elucidate the association between *Hg. leucocelaenus* and environmental features, the counts of larvae in tree holes were included as the dependent variable in GLMs with a negative binomial distribution. This distribution was preferred over the traditional Poisson distribution, because the data exhibited overdispersion (i.e., the variance was larger than the mean). The consistency of data with the negative binomial distribution was verified using the goodness-of-fit test “Minimum chi-squared” (Pearson’s chi-squared = 73.3, df = 66, *p*-value = 0.25) from the “goodfit” command from the “vcd” R package [34]. This was also confirmed by the Pearson’s chi-squared test and Dispersion Statistic ~ 1 in fitted GLMs, calculated using the R package “msme” [35]. Model selection and validation were performed as mentioned for logistic regression, with DBH, DOP, DEP, CV, HAG, RAINFALL, and OTHER_SP as the independent variables. The pseudo-R-squared for the best GLM was calculated as the proportion of deviance explained by the model using the “Dsquared” function from the “modEvA” package [32]. 

The spatial association between the distribution of the geographic location of 127 tree holes with water and the number of Culicidae larvae and eggs was evaluated using Gi* spatial statistics [36,37]. The analysis considered the number of Culicidae larvae and eggs in tree holes, including the self-points. Every 1 m was analyzed until a maximum distance of 4000 m was reached among the tree holes. We used the conditional permutation (Nsin = 999) and ran the analysis in the “spdep” package version 1.1-8 [38] in R version 4.1.0 [29]. A significance level of 5% was used, which corresponded to the minimum value of the Gi* spatial statistics at 3.2889 (N > 100 cells) according to [36]. The geographic coordinates of the points of the tree holes and the influence radius (using the upper limits of distances considered significant) obtained with the Gi* statistics allowed for identification and mapping of the clusters of tree holes in the vicinity of urban areas. This information was imported in QGIS version 3.16 [39], and nearby clusters were concatenated.

In addition, the relationship between the distribution of positive and negative tree holes with water was evaluated using Ripley’s K12 function [40]. This procedure was performed using the limits of three census sectors [41] with coordinates in UTM format as the border of the study area. We ran this analysis in R version 4.1.0 [29] and Splancs package version 2.01-42 [42] with 99 simulations and 100 repetitions. The radius of influence was obtained using Ripley’s K12 function. This information and the coordinates of the positive tree holes’ locations were used to estimate the kernel density estimation map—a procedure performed with the package “Splancs” in R. Cartographic information (e.g., land use, road and census sector limits) was obtained from the Brazilian Institute of Geography and Statistics (IBGE) and OpenStreetMap (OSM) [41,43,44,45].

## 3. Results

### 3.1. Mosquito Larval and Egg Collection

We surveyed 154 tree holes distributed from the vicinity of the ground to a height of 4.2 m in 141 trees. In this survey, 40 positive tree holes were sampled fortnightly from August 2017 to July 2018. We performed a second survey in 39 higher tree holes (from 4.3 m to 15 m high) from March to July 2018. In this second survey in the canopies, eight tree holes were identified as being positive.

A total of 1316 larvae belonging to eight species were identified as *Hg. leucocelaenus* (*n*= 698), followed by *Culex (Culex) dolosus* (*n* = 234 larvae), *Aedes argyrothorax* (*n* = 191), and *Aedes terrens* (*n* = 177). Other species corresponded to less than 1% of the total number of individuals: *Culex (Carrolia) iridescens* (*n* = 1), *Wyeomyia aporonoma* (*n* = 1), *Wyeomyia* spp. (*n* = 6), *Sabethes* spp. (*n* = 1), *Shannoniana fluviatilis* (*n* = 1), and *Toxorhynchites theobaldi* (*n* = 6). The two last species are obligate predators while the genuses *Wyeomyia* and *Sabethes* are facultative predators. All of them were found in tree holes with average volumes higher than 250 mL. Voucher specimens were deposited in the FSP Entomological Collection, USP (SI).

Additionally, we tested whether the removal of the water with eggs, larvae, and debris samples influenced the subsequent larval sampling of *Hg. leucocelaenus* during the wet and dry seasons ($\chi^{2}$_obs_ = 1.0 > $\chi^{2}$_critical_ = 3.8, *p* > 0.05, df = 1). No sample bias was found.

### 3.2. PCA and GLM Regression Analysis

**Figure 2:**
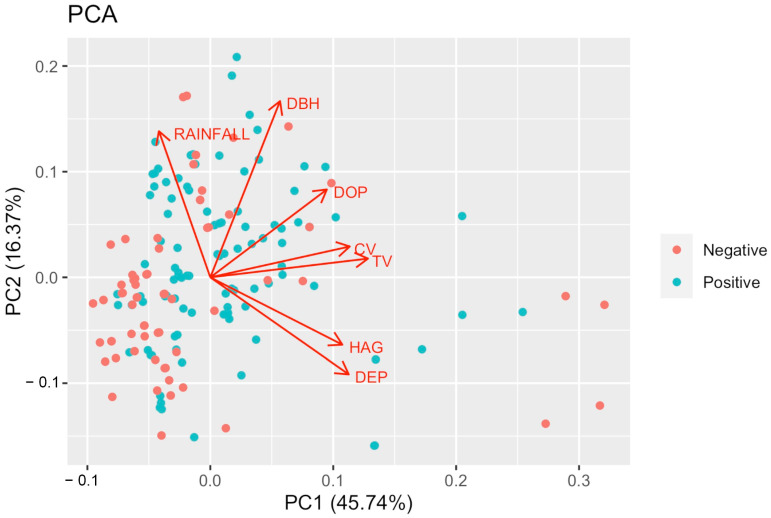
Principal component analysis of tree holes. Each point corresponds to a tree hole sampled and colored according to the classification as positive or negative for *Hg. leucocelaenus* larvae.

The following statistical analyses were performed using data collected from 173 tree hole samples. The 105 and 68 positive and negative samples analyzed for *Hg. leucocelaenus* larval presence, respectively, were from the same tree holes throughout the survey. According to PCA, the first two axes explained 62.1% of the total variation. The first axis accounted for 45.7% of the data variance, with THV and CV as the variables that contributed the most to explaining the variation in the dataset (Figure S1A). For the second component, which accounted for 16.4% of the data variance, DBH and RAINFALL were the variables that contributed the most (Figure S1B). No discrimination was noted between positive and negative habitats for *Hg. leucocelaenus*, suggesting that these tree hole categories do not exhibit specific physical characteristics (Figure 2). 

**Figure 3:**
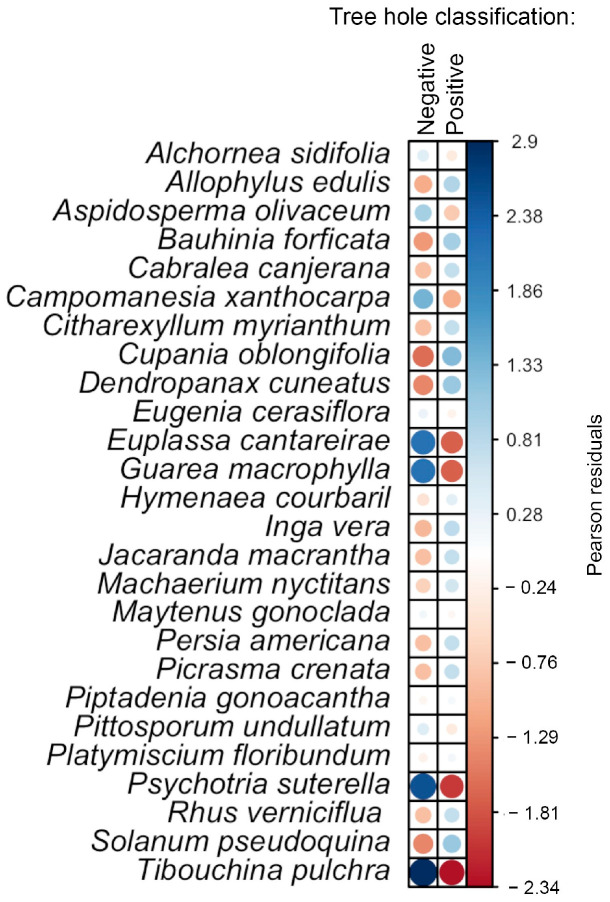
Plot of chi-squared residuals calculated for the association between tree species and the presence of negative and positive tree holes for *Hg. leucocelaenus* larvae. The circle size is proportional to the cell’s contribution to the chi-squared score. Blue indicates a positive correlation, while red indicates a negative correlation (i.e., whether a given tree species is associated with positive or negative tree holes for *Hg. leucocelaenus*).

The best model for the logistic regressions included HAG, CV, DOP, and OTHER_SP as independent variables, with a pseudo-R-squared of 0.28, indicating a good fit. Despite the PCA results, a significant association was observed between the presence of *Hg. leucocelaenus* larvae and HAG, DOP, and OTHER_SP. These results show a significant positive association (OR > 1) between *Hg. leucocelaenus* oviposition sites and the opening diameter of the tree hole (DOP). However, the presence of this species seems to be less likely (OR < 1) in tree holes occupied by other Culicidae species, or with an increase in distance from the ground (HAG) (Table 1). The distribution of tree species was significantly different between the positive and negative tree hole samples (chi-squared = 70.8, df = 25, *p* ≤ 0.001). The tree species with the highest contribution to the chi-squared score were *Euplassa cantareirae*, *Guarea macrophylla*, *Psychotria suterella*, and *Tibouchina pulchra*, all of which had increased frequency in negative samples (Figure 3).

The best GLM with negative binomial distribution adjusted for *Hg. leucocelaenus* larval count included RAINFALL, CV, DOP, and DEP as environmental independent variables, with a pseudo-R-squared of 0.14. The model indicated that *Hg. leucocelaenus* larval abundance was significantly correlated with CV and DOP, suggesting more larvae in tree holes with increased water volume and a large opening diameter. In contrast, *Hg. leucocelaenus* decreased with DEP, indicating low numbers of larvae in deeper tree holes (Table 2). 

### 3.3. Spatial Analysis of Tree Holes

**Figure 4:**
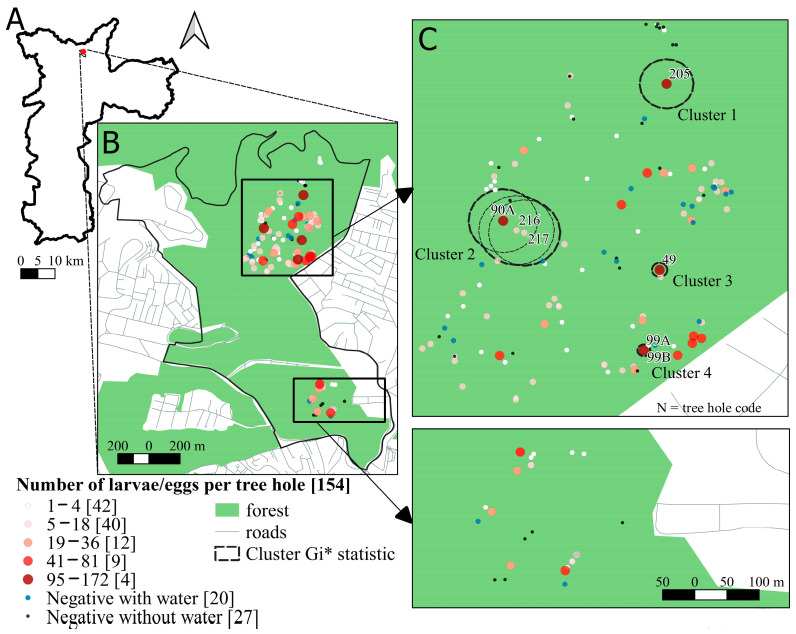
(**A**) Municipality of São Paulo; (**B**) study area; (**C**) tree hole distribution with larvae or eggs per number/tree hole and significant clusters in the Gi* statistics of numbers of larvae or eggs in tree holes at geolocated points. Numbers of larvae and tree holes are shown in the legend.

**Figure 5:**
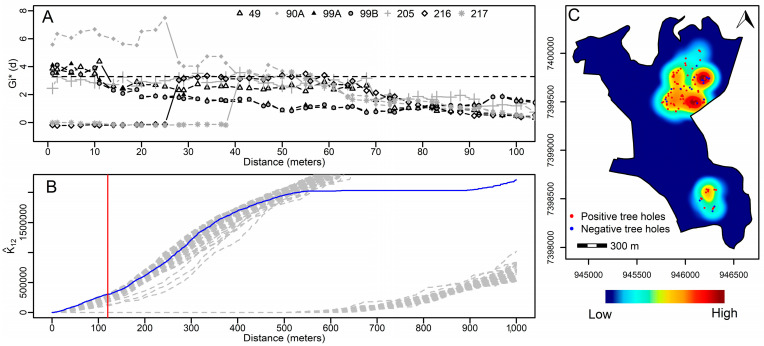
(**A**) Graph of significant clusters in the Gi* statistics of numbers of larvae around geolocated positive tree holes. (**B**) Graph of Ripley’s K-12 function analysis from positive and negative tree hole points. The blue curve above the envelope and 100 repetitions in grey show a positive spatial dependence among the sampled tree holes; the red line shows the dependence limit up to 120 m. (**C**) Kernel density map (120 m radius of influence) showing the distribution of positive tree holes and superimposed on this map; negative and positive tree holes with water are shown.

We used the Get-Ordis spatial analysis tool to identify areas with a high risk of sylvatic yellow fever in the urban forest borders. Of the 127 tree holes selected, 107 were positive for the occurrence of *Hg. leucocelaenus* and other Culicidae species, while 20 were negative. These numbers included positive and negative samples from the same tree holes as a result of the fortnightly survey. We estimated four significant clusters from the eggs and larvae of mosquito species in the study area (Figure 4C), where *Hg. leucocelaenus* co-occurs with *Ae. terrens* and *Cx. dolosus* (Table 3). It is noteworthy that the tree holes’ volumes were large enough to allow two species to co-occur in the same cavity. The volume is a measure of the size correlated with the hole’s surface area. Moreover, we identified 82 tree holes with eggs and larvae of *Hg. leucocelaenus*, ranging from 1 to 18 individuals (Figure 4B). The results of Ripley’s K12 function indicated a positive spatial dependence up to approximately 120 m (in all 100 repetitions with 99 simulations) between positive and negative tree holes with water, despite being just above the envelope line (Figure 5A). The kernel map shows that the areas with high distribution of positive tree holes are the same areas found in the GI* statistics in the northern part of the study area (Figure 5C).

### 3.4. Tree Species Identification and Positive Tree Holes

We conducted four expeditions to identify the tree species. Tree species identification was confirmed at the Dom Bento José Pickle Herbarium of the Instituto de Pesquisas Ambientais. We sampled Culicidae eggs and larvae in individual tree holes as high as 4.2 m in tree species. Even after sample collection, searches for eggs and larvae were conducted fortnightly in the same individual trees. We tested whether tree species were equally distributed between positive (*n* = 105) and negative (*n* = 68) tree holes for *Hg. leucocelaenus*. Negative tree holes were mainly distributed in *Euplassa cantareirae*, *Guarea macrophylla*, *Psychotria suterella*, and *Tibouchina pulchra* (Figure 3), and the same variables (HAG, DOP, and OTHER_SP) exhibited significant effects on larval occurrence in the logistic regression (Table 1).

We observed that some *Hg. leucocelaenus* females oviposited in tree holes from which eggs and larvae were previously removed. We estimated that most (69%) of these cavities did not exceed 250 mL.

## 4. Discussion

Previous studies on larval seasonality have frequently recorded *Hg. leucocelaenus* oviposition in ovitraps [46]. In our preliminary results, high larval density peaks of *Hg. leucocelaenus* occurred in August 2017, December 2017, and July 2018 (Figure S2A), during the humid season, coinciding with the onset epizootic cases of yellow fever in the municipality of São Paulo [2]. We sampled 59% of the total Aedini eggs in the dry season (Figure S2B), showing the effect of rainfall’s absence on the Aedini population. The quiescent eggs are an Aedini survival strategy [16]. 

Records showing oviposition in the same tree holes with volumes under 250 mL suggest that *Hg. leucocelaenus* oviposited in low volumes, which might indicate a competitive advantage owing to its rapid development. In addition, we suggest that *Hg. leucocelaenus* females revisited the tree holes suitable for oviposition. This behavior might explain the unbiased results in the sampling removal and the identified clusters of tree holes. We observed a significant association between *Hg. leucocelaenus* and the DOP of the tree hole. From the spatial analysis, DOPs varying between 8.0 and 29.0 cm were observed in three of four clusters of the tree holes in which larvae of *Hg. leucocelaenus* were distributed in large tree holes with other species (Table S1). We suggest that large volumes may be influenced by large DOPs. The clusters from spatial analysis with Gi* had large volumes and showed a potential risk of having vectors infected with yellow fever virus around the forest border. The first limitation in our project was that these observations were limited to a small number of tree holes with large volumes, so we could not confirm that this was a discernable pattern. However, we found that tree holes were mainly occupied by *Hg. leucocelaenus* as we sampled variable numbers of eggs and larvae in tree holes in the monitored area.

There was a significant association between *Hg. leucocelaenus,* and CV, which is consistent with the prediction by [47] that large tree holes tend to offer more food resources and space to support more individual insects.

We estimated *Hg. leucocelaenus* larval density at varying tree hole heights, volumes, and depths to obtain a preliminary overview of their effects. Previous descriptive analysis showed that tree holes were occupied by mosquito larvae, regardless of their position on the tree along the height gradient. Our observations of the oviposition behavior of *Hg. leucocelaenus* along the height gradient are in accordance with previous investigations suggesting that females oviposited at different levels in the Atlantic Forest [13,15]. The logistic regression analysis showed that *Hg. leucocelaenus* occurred mainly at low heights (<4.2 m) from the ground. This might be because we sampled many positive tree holes at this level. In addition, tree canopies sampled on the border of Cantareira Park were humid or had standing water in tree holes, in contrast to the canopy in Panamá forests, where water temperatures and desiccation might reduce richness and abundance with increasing height of the holes [12,48,49]. The majority of tree holes’ depths varied from 2 to 15 cm; however, the average volumes of positive tree holes at the urban forest borders were lower than 244.7 mL—large enough to nourish the larvae of two Culicidae species. With an increase in height, the model showed a lower probability of *Hg. leucocelaenus* occurring with other Culicidae species in tree holes.

The second limitation in our project is that water physicochemical variables were not measured. The physicochemical characteristics of water are of paramount value to understand Culicidae females’ choice of tree holes [47,50], and *Hg. leucocelaenus* females may have restricted their oviposition behavior, possibly by choosing containers with similar physicochemical features in the forest habitat. Moreover, PCA analysis indicated no differences between the physical characteristics of the positive and negative tree holes. Despite this, the majority of positive tree holes with low volumes supported a small number of eggs and larvae. In addition, our results showed a tendency for Culicidae abundance to increase in container habitats when water volumes were large. Nevertheless, *Hg. leucocelaenus*’s restricted habitat may impose briefer oviposition searches, although they may use olfactory and visual stimuli to identify potential oviposition sites [51].

The Ripley function analysis showed a positive tree hole surrounded by negative tree holes limited by a 120 m radius. The scale of clustering is the radius of the geographic unit in which the spatial variation in the tree holes is estimated. The small number of samples was insufficient to identify a trend and spatial dependence between negative and positive tree holes. However, this scale can be helpful in defining spatial limits to avoid contact between humans and vectors. In our study, the clusters’ distance from the domiciles varied from 64.0 to 389.9 m (Figure S3). Additionally, *Hg. leucocelaenus* was collected 5.7 km [52] from the point of release. Therefore, contact was unavoidable, and an immunization plan was implemented in the districts of the area.

In conclusion, our study increases the understanding of the relationship between *Hg. leucocelaenus* larval abundance and the physical characteristics of tree holes on the border of the Atlantic Forest. Our results show that water volume may predict *Hg. leucocelaenus* abundance, and that suitable tree holes occur throughout the height gradient. We wish to emphasize the need to address the lack of knowledge concerning the ecology of *Hg. leucocelaenus* in tree holes, which is necessary to mitigate the potential of wild yellow fever in urban forests owing to human movement.

## Figures and Tables

**Table 1:** Results of the generalized linear model (logistic regression) for the presence of *Hg. leucocelaenus* in tree holes.

Term	Estimate	SE	OR	OR 95% CI	z-Value	*p*-Value
Intercept	−0.79	0.36	-	-	−2.21	0.027
HAG	−0.002	0.000	0.998	0.996–0.999	−2.60	0.009
CV	0.001	0.000	1.00	1.00–1.00	2.07	0.04
DOP	0.23	0.05	1.27	1.15–1.39	5.01	<0.001
OTHER_SP (YES)	−3.03	0.58	0.05	0.02–0.15	−5.18	<0.001

Best GLM: *Hg. leucocelaenus* presence −HAG + CV + DOP + OTHER_SP. Pseudo-R-squared of McFadden = 0.28. SE: standard error. OR: odds ratio. CI: confidence interval.

**Table 2:** Results of the generalized linear model (negative binomial) for *Hg. leucocelaenus* larval abundance in tree holes.

Term	Estimate	SE	95% CI	z-Value	*p*-Value
Intercept	0.26	0.47	−0.82–1.37	0.57	0.57
RAINFALL	0.003	0.002	0.00–0.01	1.49	0.14
CV	0.001	0.000	0.001–0.002	4.31	<0.001
DOP	0.09	0.02	0.04–0.14	3.88	<0.001
DEP	−0.10	0.03	−0.17–−0.03	−3.30	<0.001

**Table 3:** Significant clusters from statistical analysis GI* around tree holes’ coordinates. Legend: Leuco = *Hg. leucocelaenus*, Arg = *Ae. argyrothorax*, Ter = *Aedes terrens*, Dol = *Cx. dolosus*, Toxo = *Tx. theobaldi*.

	Tree Hole ID	Eggs (N)	Leuco	Arg	Ter	Dol	Toxo	Latitude	Longitude	Range (m)
1	205	8	0	0	87	0	0	−46.634805	−23.4496659	13–42
2	90A	1	105	0	52	14	0	−46.637292	−23.4517629	1–54
	216	9	0	0	0	0	0	−46.637089	−23.4519079	33–59
	217	13	0	0	0	0	0	−46.636969	−23.4519449	43–56
3	49	0	98	0	0	20	1	−46.634911	−23.4525129	1–12
4	99A	2	6	0	0	155	0	−46.6351513	−23.4537455	1–10
	99B	2	5	0	0	0	2	−46.6351512	−23.4537455	1–10

## Data Availability

The data presented in this study are in FigShare at [https://doi.org/10.6084/m9.figshare.22684945]. They are on embargo until 2023-05-24 but they can be available on request.

## Supplementary Materials

The following supporting information can be downloaded at: https://www.mdpi.com/article/10.3390/tropicalmed8070337/s1, Figure S1—Contribution of each physical parameter to the first (A) and second (B) components of the PCA. The red dashed line shows the expected average contribution; if the contribution of the variables was uniform, the expected value would be 1/7 variables, i.e., 14.7%. Figure S2A—*Hg. leucocelaenus*’s seasonal larval density is shown in grey bars, and rainfall is shown in black lines at the southern Atlantic Forest edges (data from tree hole collections performed up to height of 4.2 m). Figure S2B—Culicidae egg distribution. Boxplots show the degree of dispersion of sampled eggs; bars show rainfall through humid and dry periods. Figure S3—Study area, with distance of significant clusters from domiciles at the forest edges. Table S1—Physical characteristics of tree holes in clusters in the vicinity of the urban area. Supplementary information (SI)—The voucher specimens of the project were deposited in the Entomological Collection of Reference of the Faculdade de Saúde Pública of the Universidade de São Paulo, labelled from numbers E-1681 to E-16296.
