# Supplementary figures and images for: Physical Attributes of Tree Holes in the Atlantic Forest Edges: Evaluating Their Association with the Presence and Abundance of Immature Haemagogus leucocelaenus
Source: Trop Med Infect Dis. 2023 Jun 25;8(7):337. doi: 10.3390/tropicalmed8070337 (PMC10383151; doi:10.3390/tropicalmed8070337)

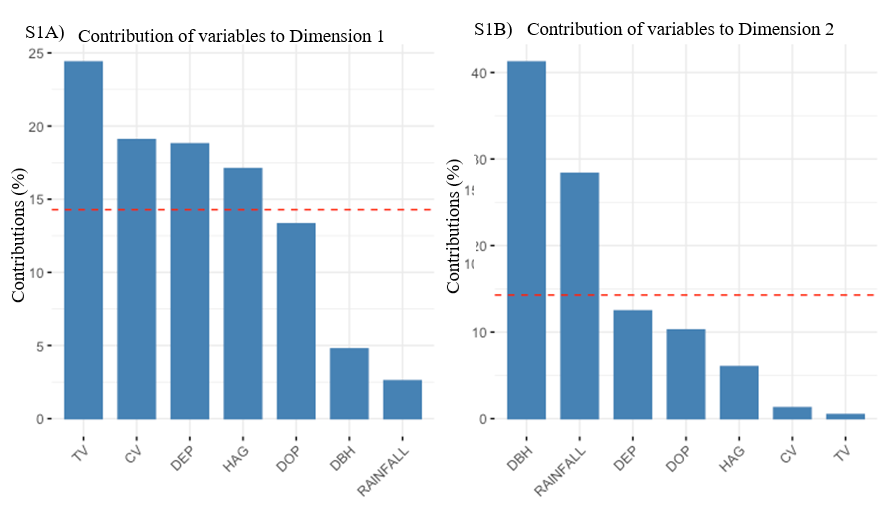

Supplement: Supplementary file 1 [file tropicalmed-08-00337-s001.zip › Figure_S1.tiff]

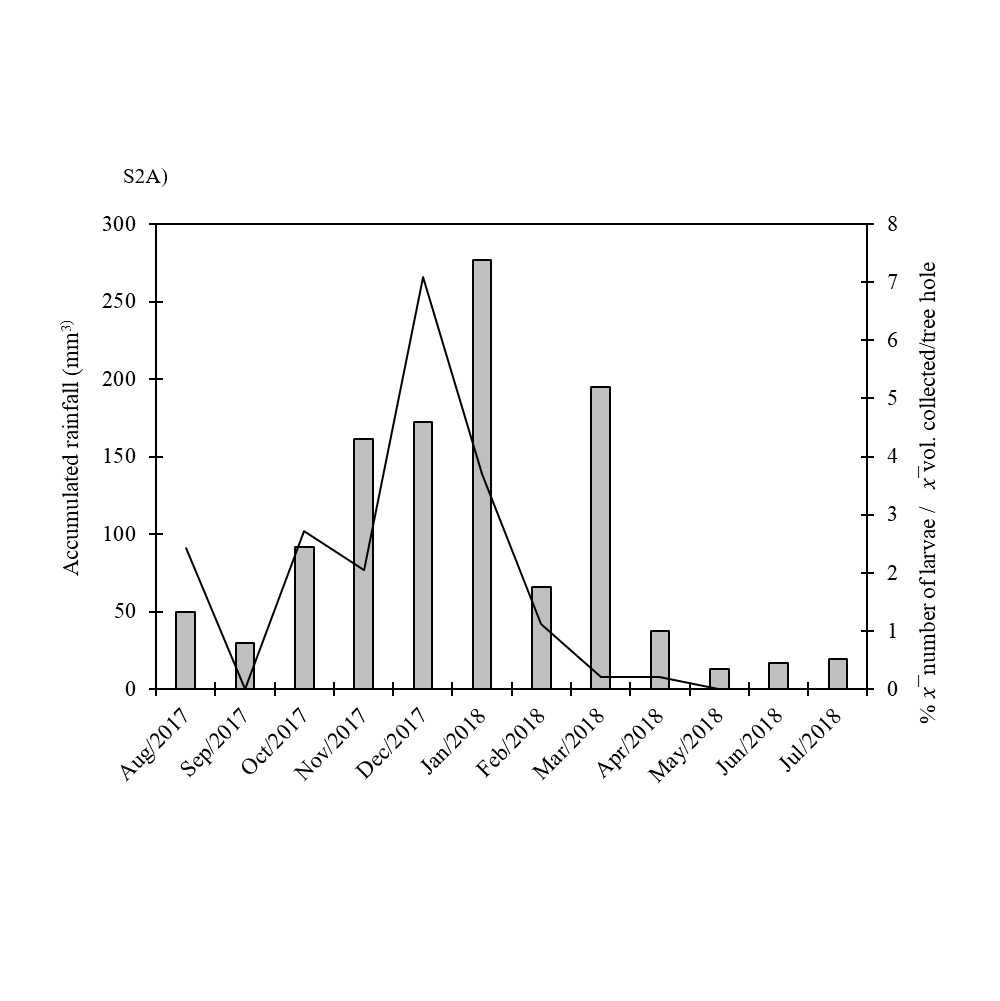

Supplement: Supplementary file 1 [file tropicalmed-08-00337-s001.zip › Figure_S2A.png]

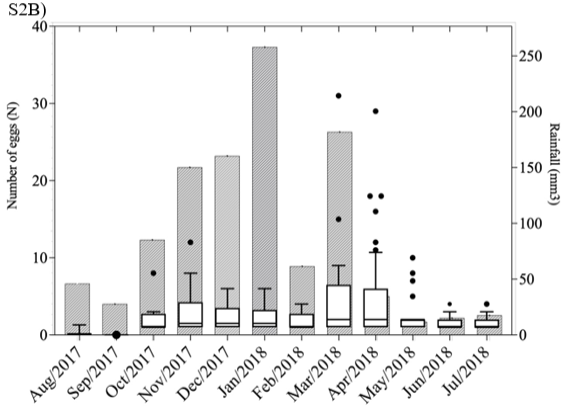

Supplement: Supplementary file 1 [file tropicalmed-08-00337-s001.zip › Figure_S2B.tiff]

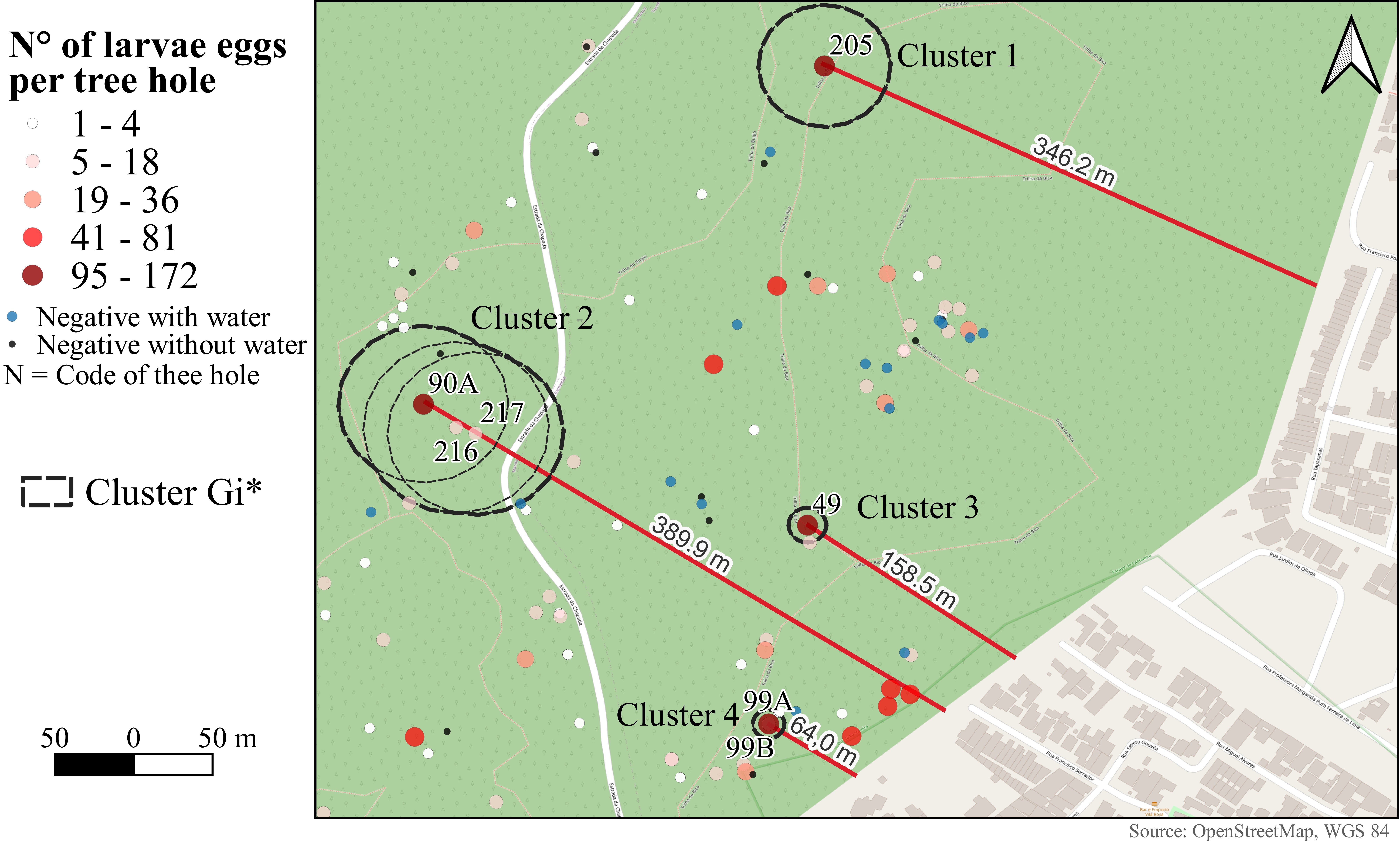

Supplement: Supplementary file 1 [file tropicalmed-08-00337-s001.zip › Figure_S3.jpg]
